# Supplementary material for: Deciphering cross-cohort metabolic signatures of immune responses and their implications for disease pathogenesis
Source: Mol Syst Biol. 2025 Sep 10;21(11):1581–98. doi: 10.1038/s44320-025-00146-w (PMC12583525; doi:10.1038/s44320-025-00146-w)
Supplement: Supplementary file 1 — Appendix [file 44320_2025_146_MOESM1_ESM.pdf]

Appendix for

Deciphering Cross-Cohort Metabolic Signatures of Immune Responses and Their Implications for Disease Pathogenesis

Jianbo Fu[1,2], Nienke van Unen[1,2], Andrei Sarlea[3], Nhan Nguyen[1,2], Martin Jaeger[3], Javier Botey-Bataller[1,2,3], Valerie A.C.M. Koeken[3], L. Charlotte de Bree[3], Vera P. Mourits[3], Simone J.C.F.M. Moorlag[3], Godfrey Temba[3,4], Vesla I. Kullaya[4,5], Quirijn de Mast[3], Leo A.B. Joosten[3,6], Cheng-Jian Xu[1,2], Mihai G. Netea[3,7,9], Yang Li[1,2,3,8,9,10*]

Table of contents

Appendix Figure S1: page2
Appendix Figure S2: page3
Appendix Figure S3: page4
Appendix Figure S4: page5
Appendix Figure S5: page6
Appendix Figure S6: page7
Appendix Figure S7: page8
Appendix Figure S8: page9
Appendix Figure S9: page10
Appendix Figure S10: page11
Appendix Figure S11: page12
Appendix Figure S12: page13

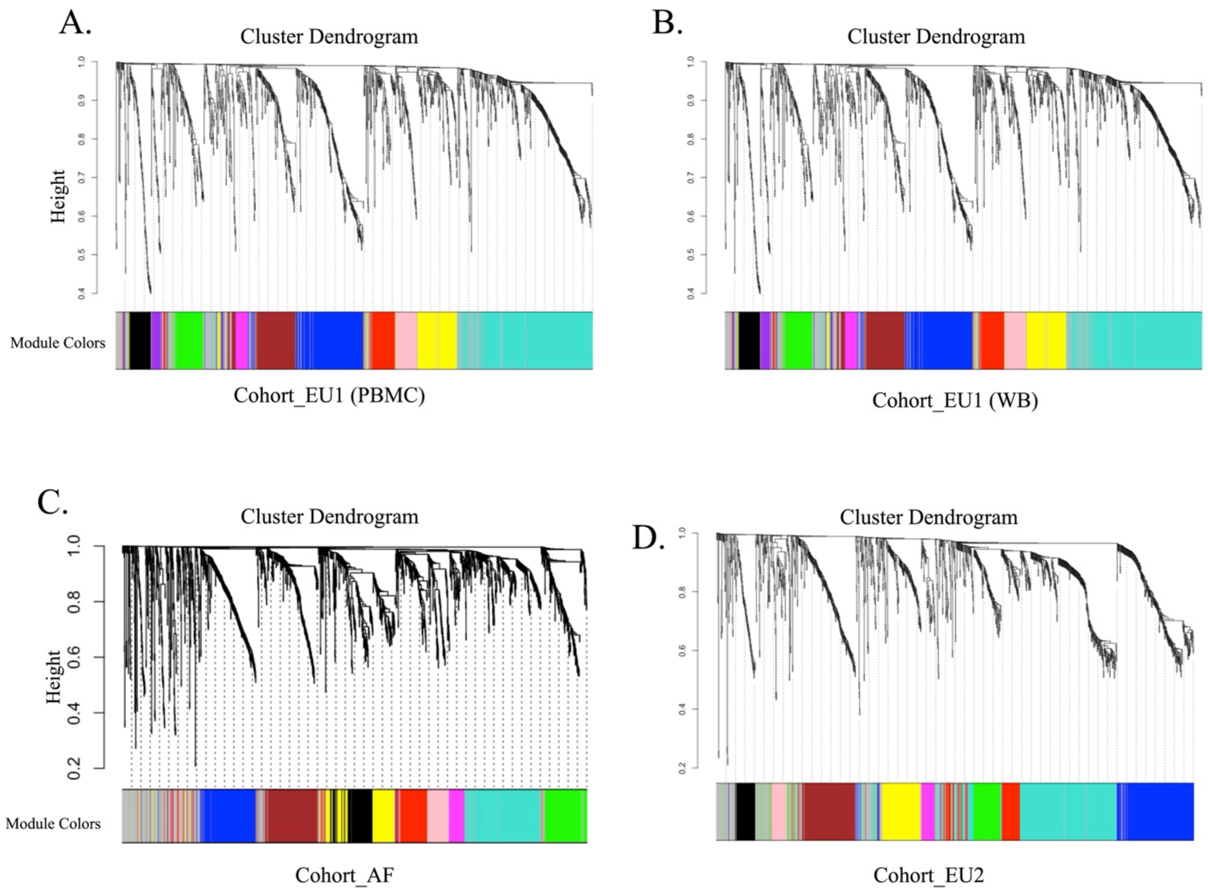

Appendix Figure S1. Hierarchical clustering dendrogram of metabolites data (a, b and c). The WGCNA analysis reveals the association of metabolite groups with cytokine responses (IL-1β, IL-6, TNF, and IFN-γ) triggered by S. aureus. The specific metabolites within each module delineated by WGCNA are detailed in the Dataset EV1.

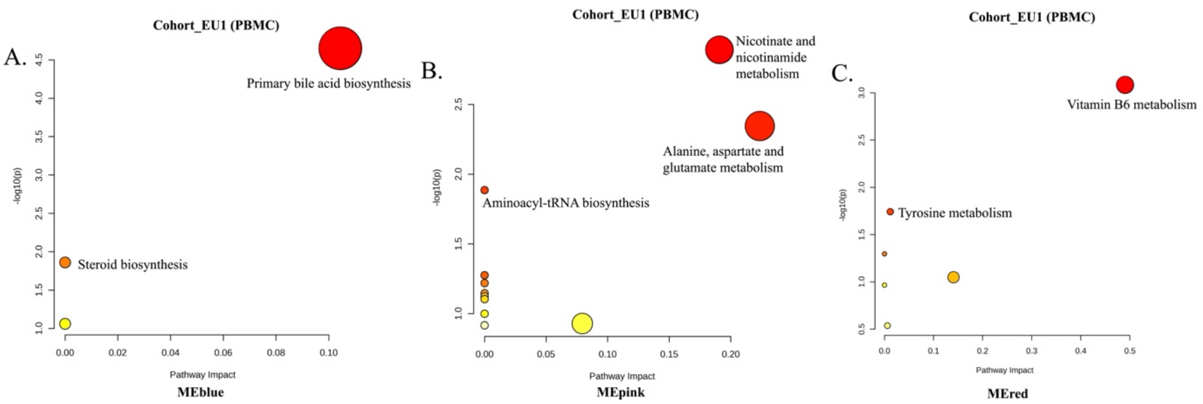

Appendix Figure S2. The pathway analysis results for metabolites modules within the Cohort_EU1 and Cohort_AF. Scatter plots illustrate the pathway analysis results for metabolites within the MEpink (Figure 2A), MEred (Figure 2A), MEblue (Figure 2A) modules of Cohort_EU1 and MEred (Figure 2B) module of Cohort_AF. The darker the bubble color, the larger the -log(Pvalue), indicating higher significance.

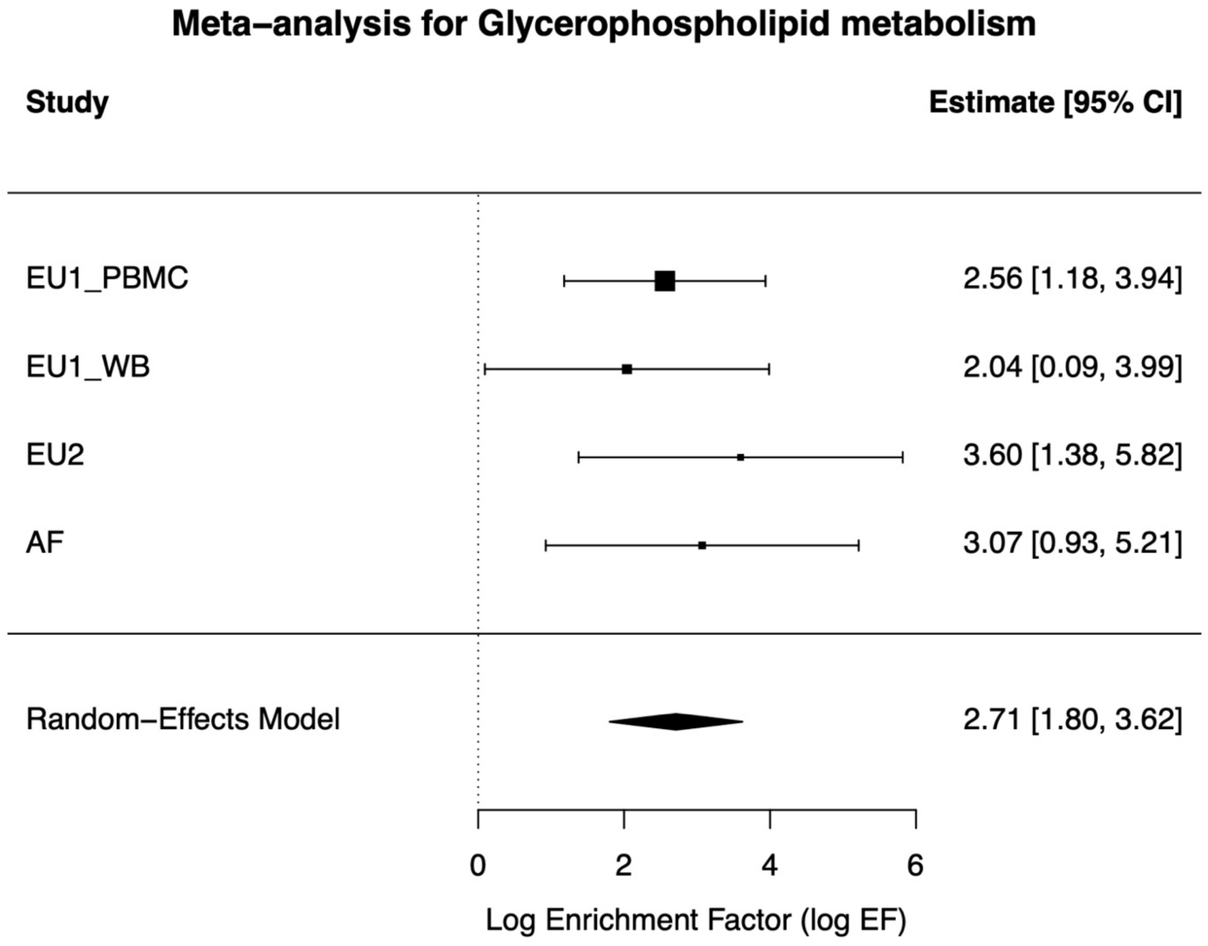

Appendix Figure S3. Meta-analysis forest plot for the glycerophospholipid metabolism pathway across four datasets (Cohort_EU1 PBMC, Cohort_EU1 WB, Cohort_EU2 and Cohort_AF). Each square represents the study-specific log enrichment factor (log EF) with its 95 % CI; the diamond shows the pooled estimate from a random-effects model (log EF = 2.71 [95 % CI 1.80–3.62]. Heterogeneity was negligible ($I^2 = 0$ %, $Q = 1.22$, $p = 0.748$).

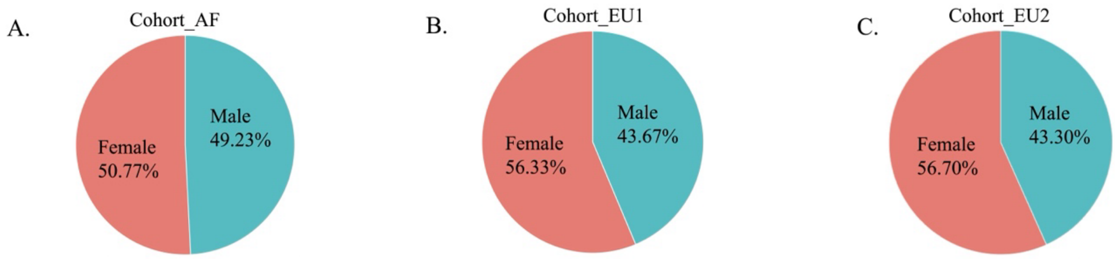

Appendix Figure S4. Cohort Sex composition of Sex differences in phosphatidylcholine/phosphatidate–cytokine associations. Panels A–C: cohort sex ratios (AF, EU1, EU2).

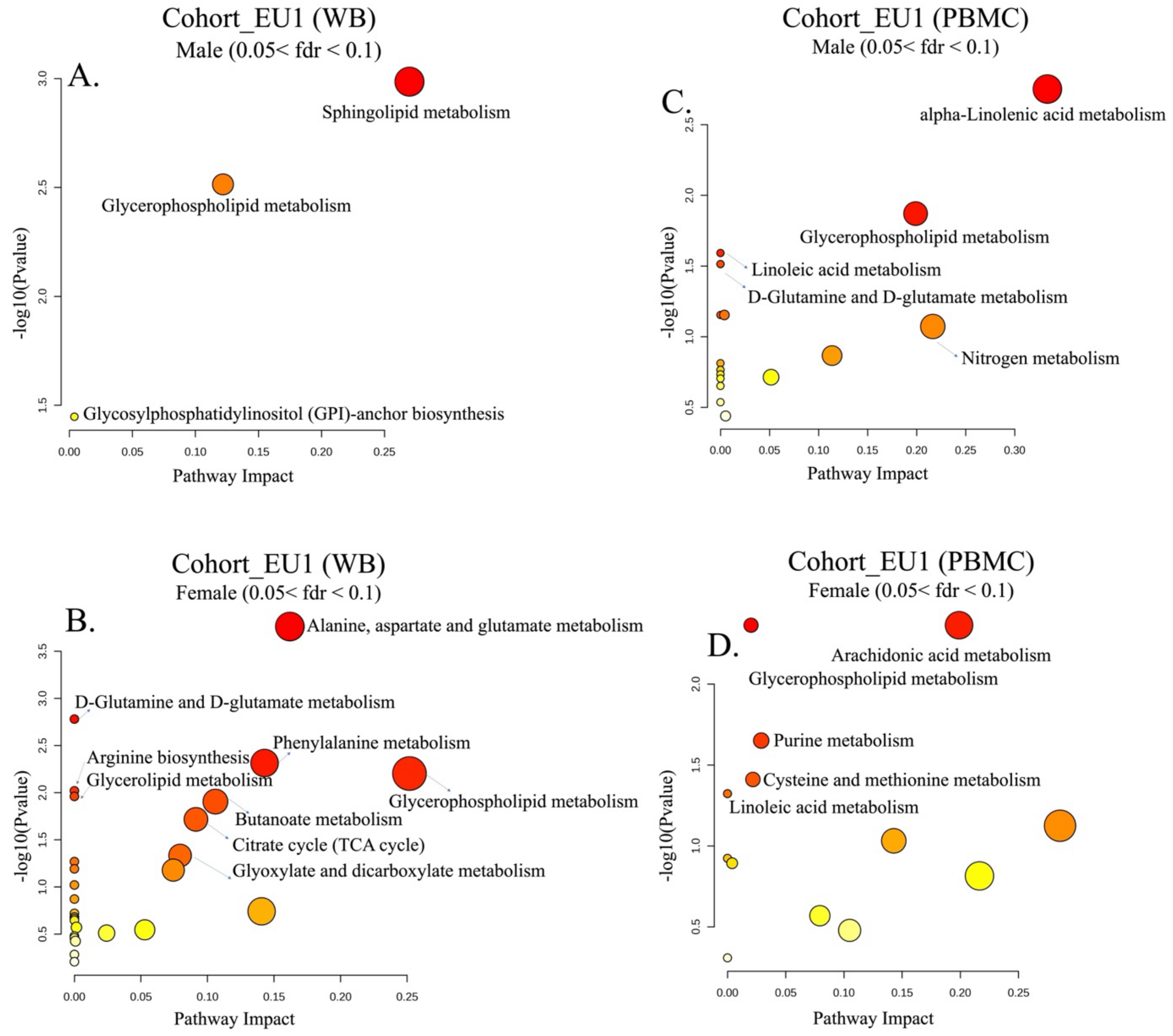

Appendix Figure S5. Scatter plots illustrate the results of the pathway analysis obtained by correlating males and females separately in Cohort_EU1 with correlated metabolites (0.05 < FDR < 0.1) with either cytokine responses (IL-1β, IL-6, TNF, and IFN-γ) induced by S. aureus stimulation. The darker the bubble color, the larger the -log(Pvalue), indicating higher significance.

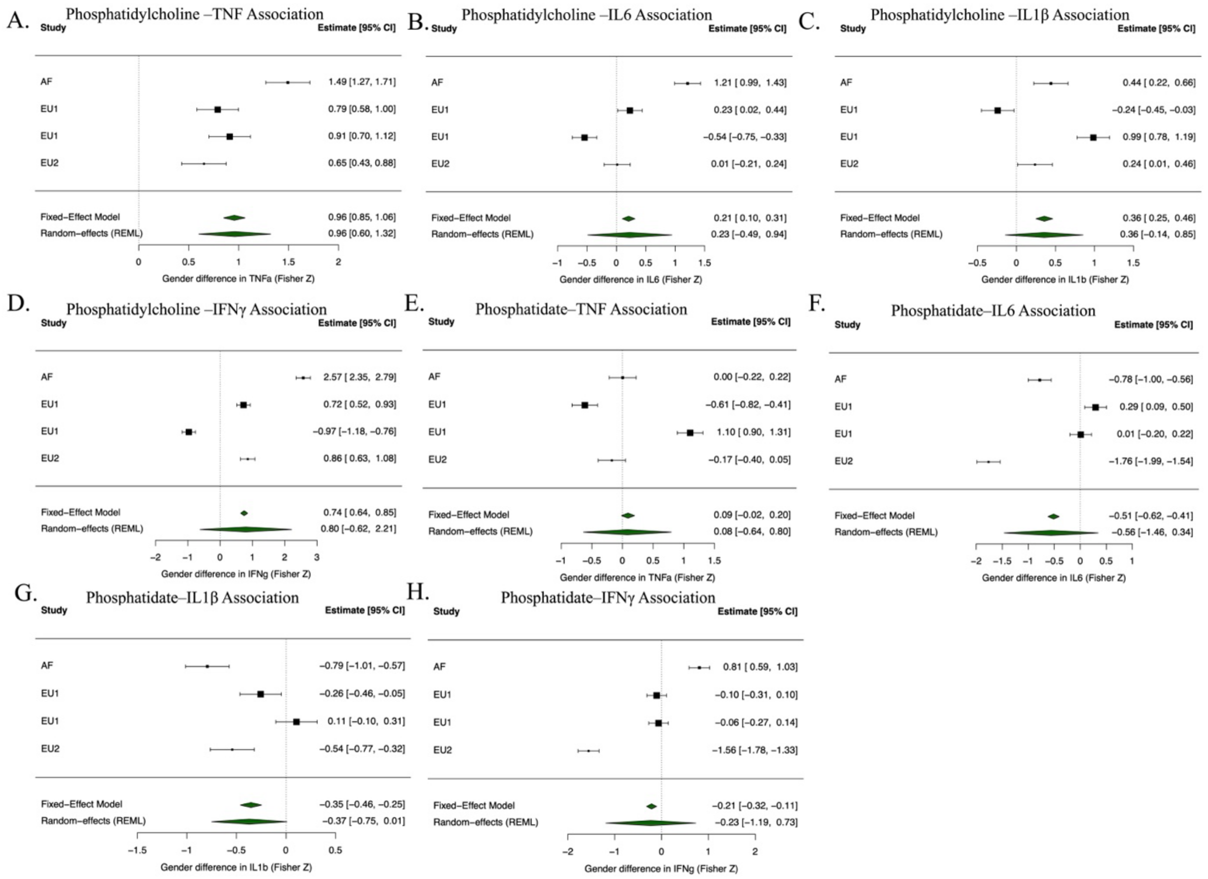

Appendix Figure S6. Cohort Sex meta-analysis of Sex differences in phosphatidylcholine/phosphatidate–cytokine associations. A–H: forest plots of gender differences (male vs. female) in Fisher's Z–transformed Spearman r for lipid–cytokine pairs (A: PC–TNF; B: PC–IL-6; C: PC–IL-1β; D: PC–IFN-γ; E: phosphatidate–TNF; F: phosphatidate–IL-6; G: phosphatidate–IL-1β; H: phosphatidate–IFN-γ). Each square represents the cohort-specific gender difference estimate (male vs. female) with its 95 % CI; diamonds show the pooled effect under fixed- and random-effects models.

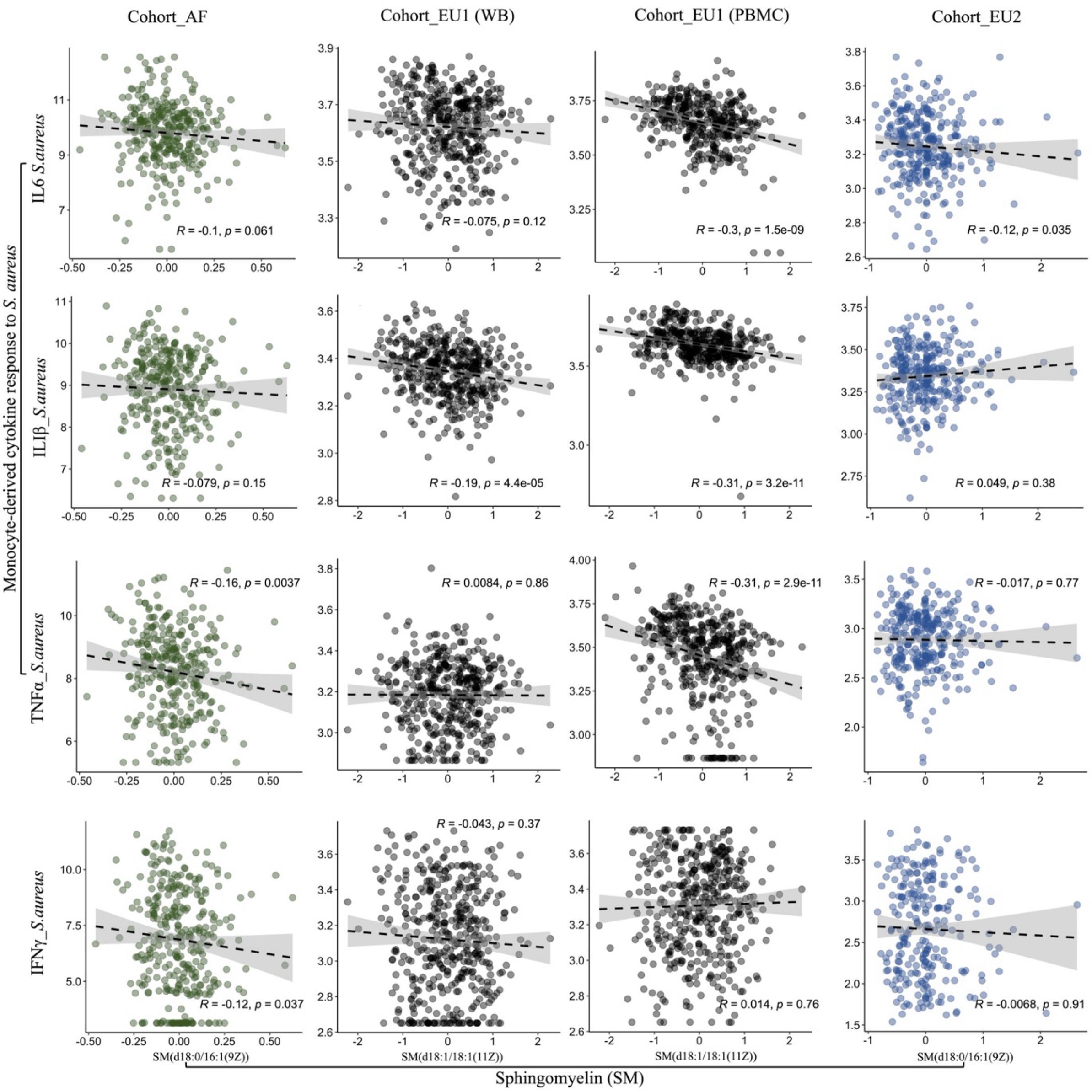

Appendix Figure S7. Linear regression between sphingomyelin (SM) at baseline and S. aureus induced cytokine responses (IL-1β, IL-6, TNF, and IFN-γ) in the Cohort_AF, Cohort_EU1 and Cohort_EU2. r: Spearman's correlation coefficient.

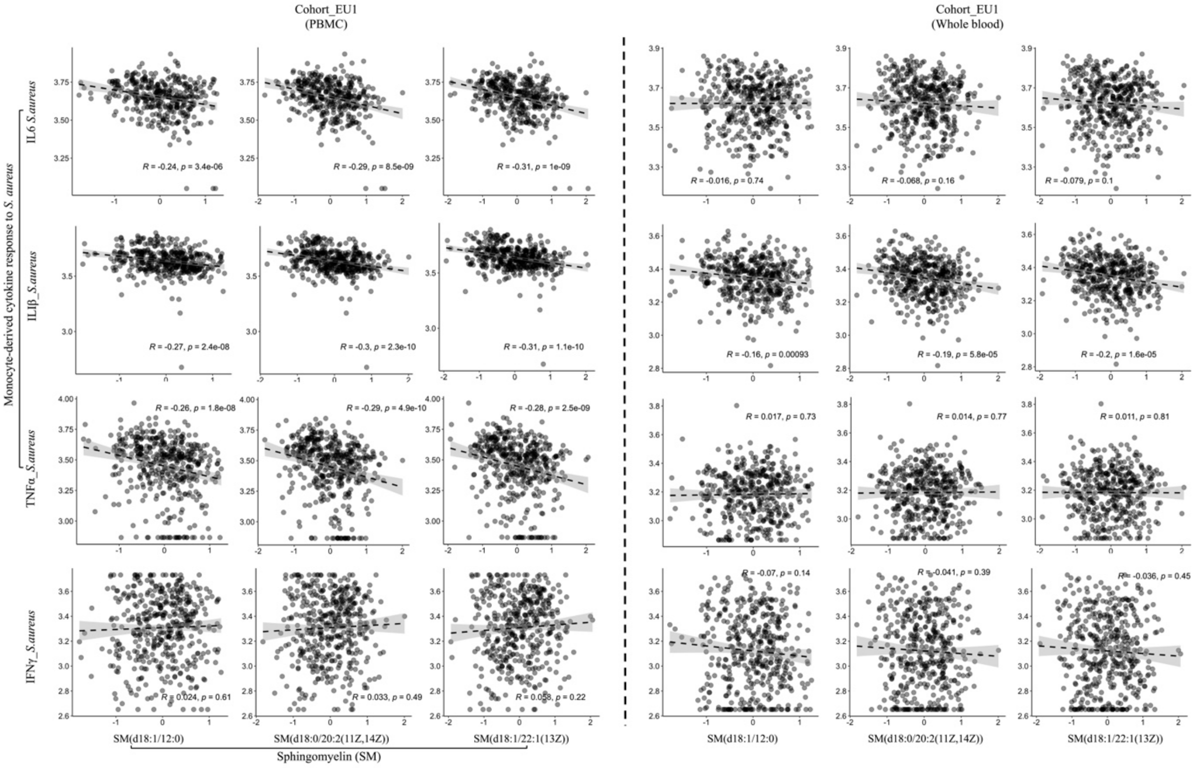

Appendix Figure S8. Linear regression between sphingomyelin (SM) at baseline and S. aureus induced cytokine responses (IL-1β, IL-6, TNF, and IFN-γ) in the Cohort_EU1. r: Spearman's correlation coefficient.

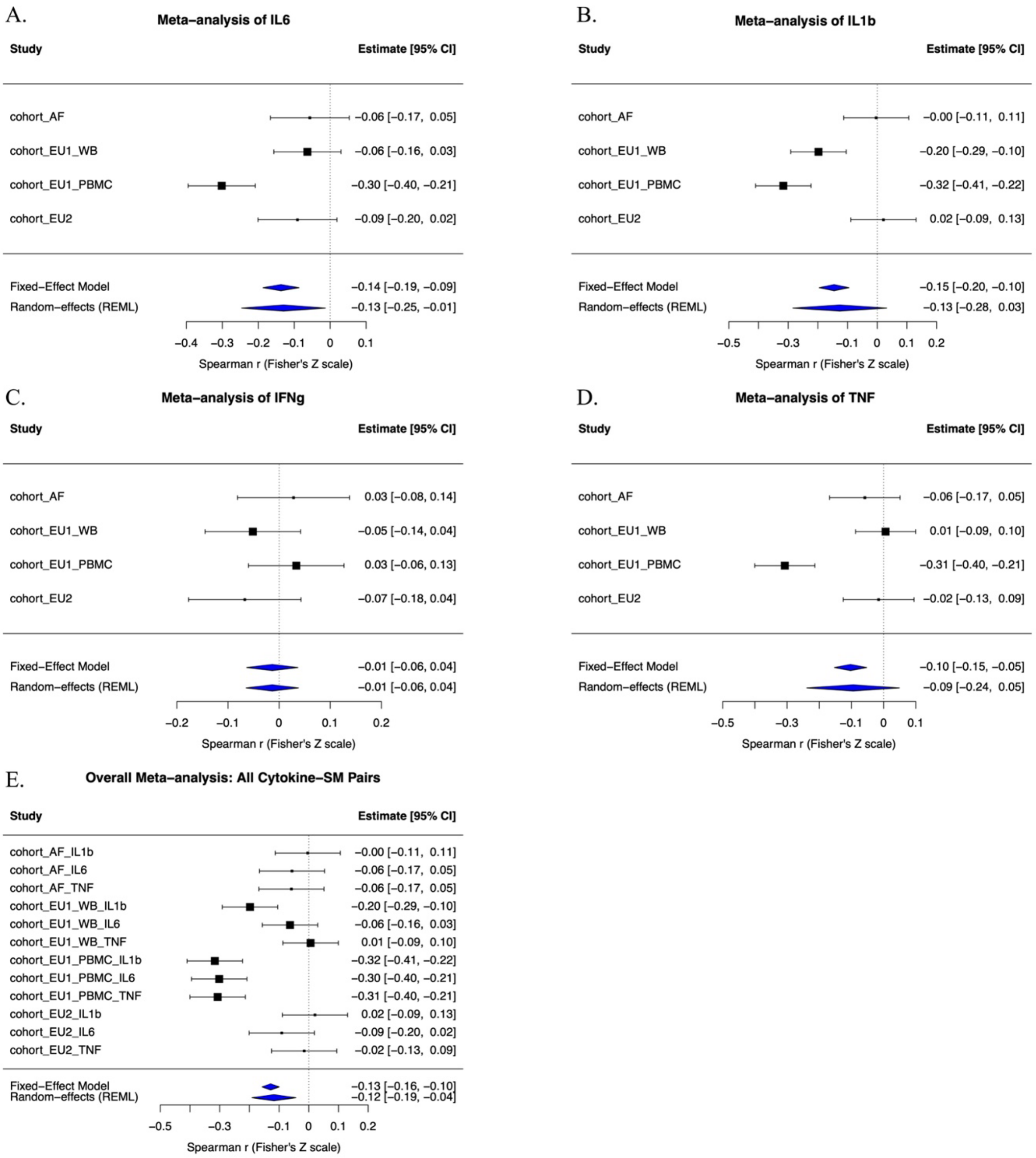

Appendix Figure S9. Forest-plot meta-analyses of sphingomyelin–cytokine correlations across four cohorts. (A–D) Cohort-specific Fisher's Z–transformed Spearman r estimates (squares) and 95 % CIs (horizontal lines) for the association between sphingomyelin levels and IL-6 (A), IL-1β (B), IFN-γ (C) and TNF (D). Diamonds show the pooled effect under fixed- and random-effects models. (E) Overall meta-analysis combining all cytokine–sphingomyelin pairs, with individual cohort estimates and the summary effect.

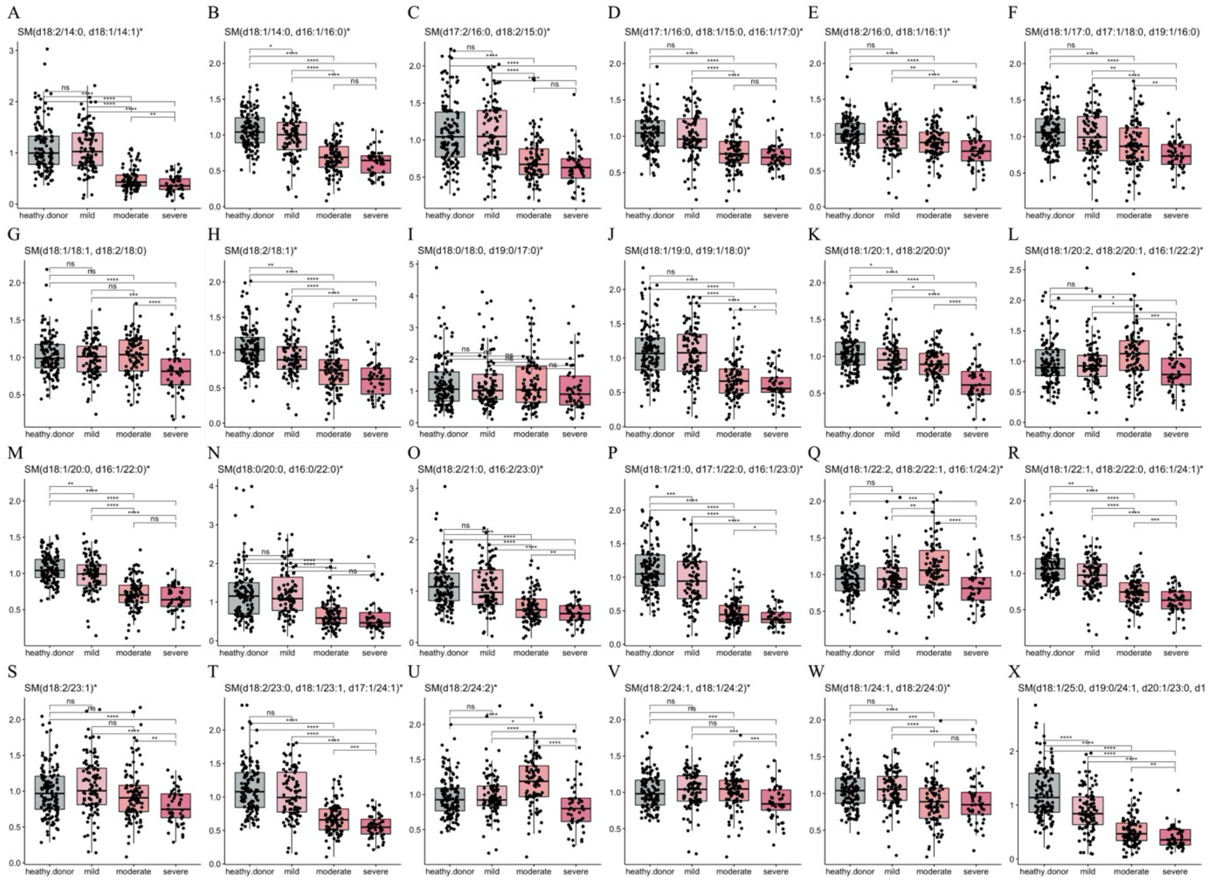

Appendix Figure S10. Changes in sphingomyelins of sphingolipid metabolism in COVID-19 patients. (A-X) Gray denotes a healthy individual while increasing shades of red indicate escalating severity of COVID-19 (*p<0.05, **p<0.01, ***p<0.001).

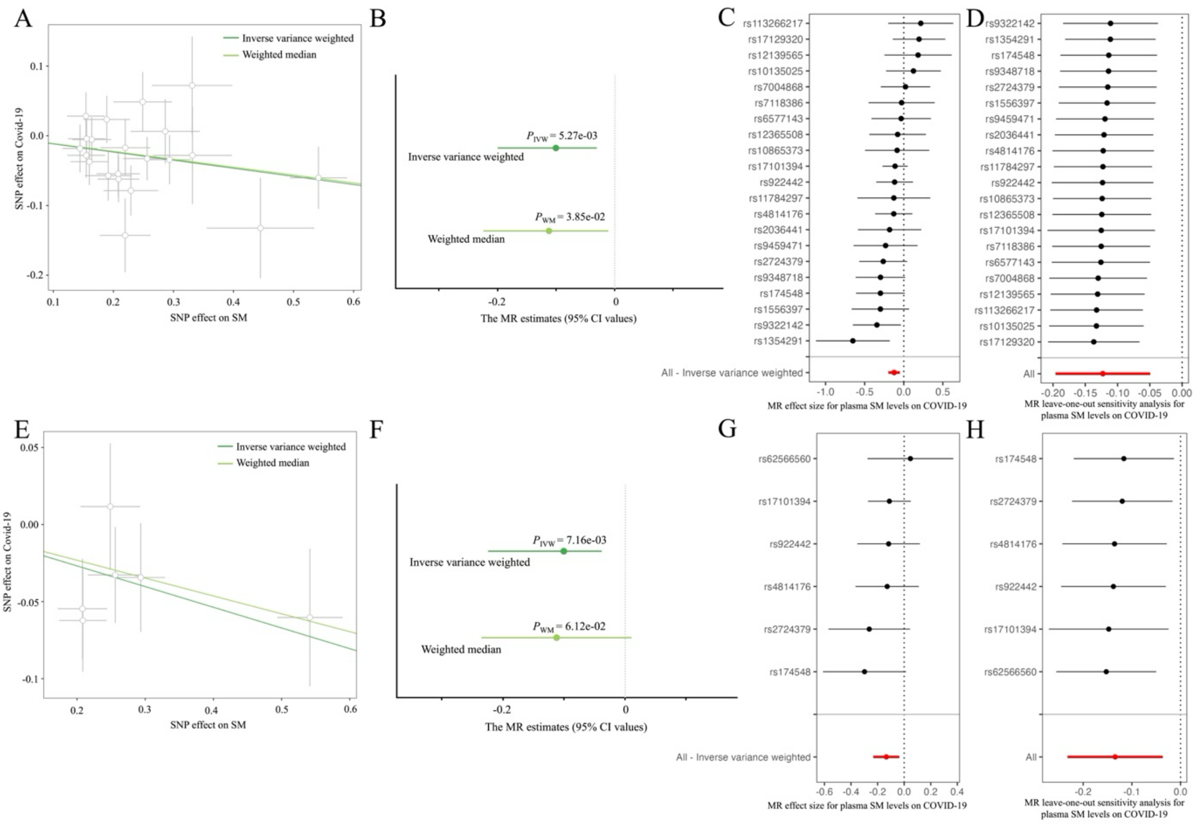

Appendix Figure S11. Mendelian randomization of plasma sphingomyelin (SM) levels on COVID-19 severity using instruments selected at two p-value thresholds. Panels A–D show results for SNPs with $p < 1 \times 10^{-6}$; panels E–H show results for SNPs with $p < 5 \times 10^{-8}$. A, E. Scatter plots of per-SNP effects on SM (x-axis) versus effects on COVID-19 severity (y-axis). Each point represents one genetic instrument, with horizontal and vertical bars denoting its 95% confidence intervals. Dark green and light green lines show the inverse-variance weighted (IVW) and weighted-median regression slopes, respectively. B, F. Forest plots of the overall MR estimates from IVW and weighted-median methods. Dots indicate point estimates (β) and horizontal lines their 95% CIs; corresponding p-values are labeled. The vertical dashed line denotes the null. C, G. SNP-specific MR effect estimates (black dots with 95% CIs) and the overall IVW summary (red diamond at bottom). SNPs are ordered along the y-axis; the vertical dotted line marks no effect. D, H. Forest plots of MR leave-one-out sensitivity results.

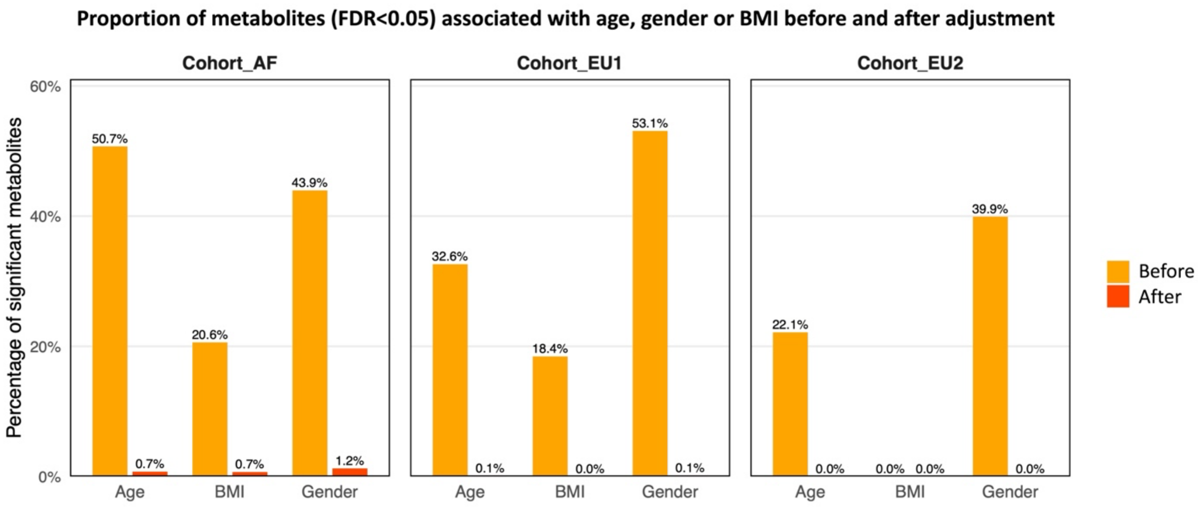

Appendix Figure S12. Proportion of metabolites (FDR < 0.05) associated with age, gender or BMI before (orange) and after (red) adjustment in the AF, EU1 and EU2 cohorts. Adjustment reduces all associations to near zero, demonstrating effective removal of these confounders.
